# Feasibility and potential effects of using the electro-dress Mollii on spasticity and functioning in chronic stroke

**DOI:** 10.1186/s12984-020-00740-z

**Published:** 2020-08-10

**Authors:** Susanne Palmcrantz, Gaia Valentina Pennati, Hanna Bergling, Jörgen Borg

**Affiliations:** Karolinska Institutet, Department of Clinical Sciences, Danderyd Hospital, Division of Rehabilitation Medicine, Entrévagen 8, SE-182 88 Stockholm, Sweden

**Keywords:** Stroke, Spasticity, Home setting, Self-perceived, Clinical assessments, Outcome, Self-administered

## Abstract

**Background:**

Spasticity after lesions of central motor pathways may be disabling and there is a need for new, cost-effective treatment methods. One novel approach is offered by the electro-dress Mollii®, primarily designed to enhance reciprocal inhibition of spastic muscles by multifocal, transcutaneous antagonist stimulation.

**Methods:**

The Mollii® suit was set individually for 20 participants living with spasticity and hemiplegia after stroke and used in the home setting for 6 weeks. Usability and perceived effects were monitored by weekly telephone interviews. Outcome was assessed by use of the NeuroFlexor™ method for quantification of the neural component (NC) of resistance to passive stretch (spasticity), and the modified Ashworth scale (MAS) for total resistance, Fugl-Meyer Assessment of motor recovery for sensorimotor function in upper (FM-UE) and lower extremities (FM-LE), activity performance with the Action Research Arm Test (ARAT), Berg balance scale, 10 m and 6 min walk tests, and perceived functioning with the Stroke Impact Scale.

**Results:**

Compliance was high (mean 19.25 of 21 sessions). Perceived positive effects were reported by 60% and most commonly related to decreased muscle tone (*n* = 9), improved gait pattern function (*n* = 7) and voluntary movement in the upper extremity (*n* = 6). On a group level, the NC decreased significantly in the wrist flexors of the affected hand (*p* = 0.023) and significant improvements according to FM-UE (*p* = 0.000) and FM-LE (*p* = 0.003) were seen after the intervention. No significant difference was detected with MAS or assessed activity performance, except for the ARAT (*p* = 0.000). FM-UE score change correlated significantly and fairly with the perceived effect in the upper extremity (*r* 0.498 *p* = 0.025) and in the corresponding analysis for the FM-LE and perceived effect in the lower extremity (*r =* 0.469 *p* = 0.037).

**Conclusion:**

This study indicates that the Mollii® method is feasible when used in the home setting to decrease spasticity and improve sensorimotor function. The results may guide a larger controlled study combined with rehabilitation interventions to enhance effects on activity and participation domains.

**Trial registration:**

NCT04076878. Registered 2 September 2019 - Retrospectively registered

## Background

Spasticity, in terms of “velocity dependent increase of resistance to passive muscle stretch” [1], is a common manifestation of “muscle overactivity” seen in spastic paresis [2] that may follow lesions of central sensorimotor pathways, such as after stroke, traumatic brain injury, in cerebral palsy or spinal cord injury and may be associated with increased impairments, activity limitations and restrict participation [3–5]. In addition to the human costs, the estimated direct costs for managing patients with spasticity after stroke are approximately four times higher than for patients without spasticity [4]. Treatment of spastic paresis is based on comprehensive physiotherapy, which may be combined with pharmacological and surgical treatments if needed. After stroke, today’s first line add-on therapy is by use of intramuscular injections of botulinum toxin A (BTX). There is consistent evidence that focal spasticity and associated disabilities after stroke may be reduced by this treatment [6–12] but also that issues remain. Recently, a systematic review by Andringa et al. [9] concluded that while treatment with BTX in the upper limb improves passive movement of spastic wrist and fingers as well as self-care, there is also a demonstrated lack of effects on arm-hand activity performance. A corresponding review of the literature on treatment of lower limbs by Gupta et al. [12] found that the evidence on effects on mobility was not robust and pointed out the need for new controlled trials. Although treatment with BTX is well established, it is not generally available, not all patients respond well and the maximal dose does not always allow treatment of multifocal spasticity.

Another treatment approach in this area is modulation of sensorimotor input by use of transcutaneous electrical nerve stimulation (TENS) which can be self-administered and is considered cost-effective, with few side effects and thus a promising alternative or complement to current standard therapies [13]. Recent, systematic reviews suggest that treatment with TENS transcutaneous may have beneficial effects on spasticity after stroke [13–15]. These findings, lend support to the new treatment method, the electro-dress Mollii®, evaluated in this study, which offers TENS to be applied at multiple stimulation points.

The Mollii® method has been developed by Inerventions AB, which is a Swedish medtech company, and represents an innovative approach for non-invasive electro-stimulation to reduce spasticity and improve motor function. The Mollii® method is provided in a tight fitting, whole body suit with multiple electrodes that can be set individually. The Mollii® method uses low frequencies and low intensities that evokes sensory input but does not elicit muscle contractions. The theoretical background of this method primarily refers to the concept of reciprocal inhibition, i.e. that sensory input from a muscle may inhibit the activation of an antagonistic muscle through activation of disynaptic reciprocal Ia inhibitory pathways [16, 17]. Thus, the application of Mollii® aims at stimulating an antagonist muscle (e.g. the anterior tibial muscle) to reduce the reflex mediated muscle over-activity in an antagonist muscle (e.g. the gastrocnemius muscle), by inducing reciprocal inhibition. However, as for conventional low intensity TENS, other mechanisms related to altered sensory input, may also play a role [13].

There is now a growing experience from pilot applications of Mollii® in patients with cerebral palsy and stroke indicating that application of this method is feasible and may have beneficial effects on spasticity related disabilities [18]. However, specific effects on spasticity and how these relate to perceived and assessed functioning and disability remain to be demonstrated.

Thus, the aims of this study were to explore the feasibility of using the Mollii® suit in the home setting for 6 weeks and to explore potential effects on functioning in chronic stroke. Specific aims were to explore the clinical relevance in terms of: 1) perceived usability 2) potential self-reported and assessed changes in spasticity and other functioning after the 6 weeks intervention and 3) if these potential changes were associated with level of functioning and 4) perceived changes in functioning.

## Methods

### Setting

This study was performed at the University Department of Rehabilitation Medicine, Danderyd Hospital in Stockholm, Sweden.

### Design and study population

Using an explorative single group design, a convenient sample of 20 participants was planned to take part in the intervention. Eligible participants had suffered a stroke ≥12 months earlier (verified by CT or MRI examination) and were living with hemiplegia affecting the right or the left side of the body including both upper and lower extremity function. They were able to walk with assistance or independently according to the Functional Ambulatory Categories [19] with a score of 2–5. Activity in upper extremity was limited according to the Action Research Arm test (ARAT) [20] but a grasp and grip movement could be voluntarily performed. Moreover, eligible study participants were > 17 years old, able to understand instructions as well as written and oral study information and could express informed consent.

Exclusion criteria comprised no detected neural component exceeding the cut off for spasticity according to the NeuroFlexor™ (> 3. 4 Newton) in the wrist flexors [21], contractures not compatible with performing the NeuroFlexor™ test, any other disorder with an impact on sensorimotor function, any other severe concomitant disease (such as cancer, cardiovascular, inflammatory or psychiatric disease), uncontrolled epilepsy or blood pressure, major surgery during the last year, any implanted medical devices, pregnancy and BMI > 35.

Participants with ongoing pharmacological treatment (e.g. with Baclofen) could be included only if the medication was stable since at least 3 months and no change during the study period was anticipated. Participants, who had been subject to intramuscular treatment for spasticity could participate only if the time since last treatment was 3 months or more and if it was anticipated that next treatment would not be given during the study period.

Eligible participants were identified in out-patient care in Stockholm, Sweden, by physiotherapists who informed the patient about the study and asked the patient for consent to be contacted by the study coordinator who obtained informed consent. First enrollment was in August 15, 2017 and study completion in February 1, 2019.

### Intervention

**Fig. 1:**
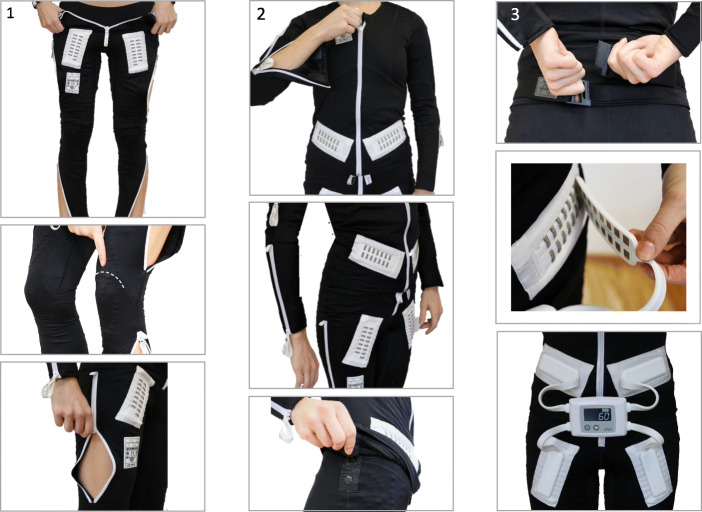
Donning of the Mollii® suit – trousers [1] and jacket [2] and connection of the control unit to the suit [3]

As the method is based in the theory of reciprocal inhibition of the spastic muscle [16, 17], the Mollii®suit (Inerventions AB, Danderyd, Sweden) was set to stimulate the antagonist of the spastic muscles by a person trained in the Mollii® method. These settings were based on the results of the assessment (presented in the data collection section) performed and reported by the experienced physiotherapist in the study who was not otherwise involved in the settings. During donning, the trousers are put on first as presented in Fig. 1. Next the user proceeds to put on the jacket and zip it up. The Mollii® suit has a tight fit to allow the electrodes to adhere to the skin surface (Fig. 1). The control units were programmed before use and connected to the suit. Clinical experience and follow-up with users have shown that the Mollii® suit have detectable effects at 20 Hz of stimulation when used every second day 60 min/session. Thus, in this study, the suits were set at 20 Hz and to stimulate for 60 min/session.

The participants were instructed in how to use the Mollii® suit and to use the suit for 60 min, every second day, for 6 weeks (21 sessions). Study participants were encouraged to continue everyday life activities as usual.

### Data collection

An experienced physiotherapist and a physician, both trained in the data collection methods performed the assessments of the participants’ functioning and disability before the intervention (M1) and repeated at the end of the 6 weeks period (M2).

#### Characteristics of participants

Characteristics were collected in terms of age, sex, diagnosis, paretic side, time to inclusion from stroke onset and independence in walking with the Functional Ambulation Categories [19] as well as independence in self-care and mobility by use of the Barthel Index [22]. Cognitive function was assessed with Montreal Cognitive Assessment [23].

#### Usability and perceived effects

**Fig. 2:**
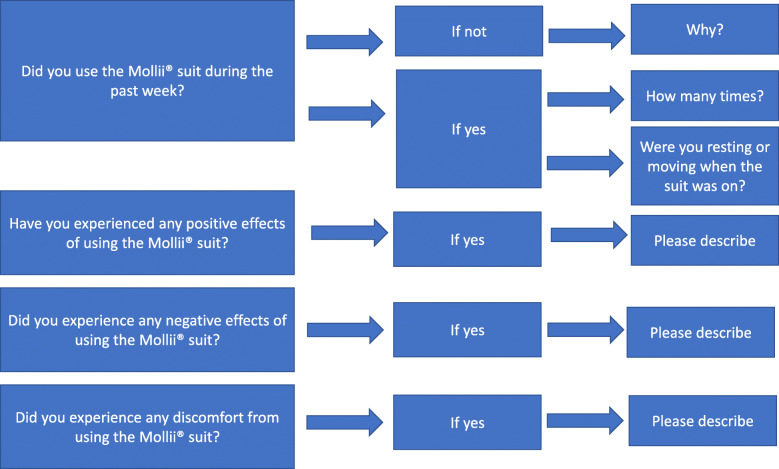
Questions asked in weekly telephone interviews

Participants perceptions of using the suit in the home setting was collected in weekly telephone interviews during the 6 weeks intervention by an experienced physiotherapist. The interview questions are presented in Fig. 2. The response to each question was recorded in a log-book by the interviewer. Perceived effects were analyzed and coded A) according to the International Classification of Functioning and Disability [24] and B) into 1) no effect, 2) potentially positive and 3) positive effect and further into C) effects in 1) upper, 2) lower extremity and 3) general effects. The coding was discussed with the data collector to assure consistency and validity. During the last telephone interview, the participants were asked to rate the overall usability of the suit on a 10-point scale ranging from 1 (no usability) to 10 (maximum usability).

#### Assessments related to spasticity

The neural component (NC), of resistance (Newton) to passive muscle stretch of the wrist flexors according to the NeuroFlexor™ method (Aggero MedTech AB, Solna, Sweden) was recorded at 3 time points (A1-A3) before the intervention to assess potential fluctuations in NC and at M1 and M2, to assess potential change over the intervention time. Further, NC was quantified before and during one test treatment session before the intervention started in order to explore immediate effects of the treatment. These tests were performed with the participant at rest, in a sitting position. The NeuroFlexor™ incorporates a neuro-biomechanical computerized model that allows quantifying and differentiating the neural reflex contribution (here defined as spasticity according to Lance [1]) and mechanical contributions to total resistance to passive muscle stretch. The validity, reliability and sensitive to change of this method have been demonstrated [25–27]. Reference data for upper limb have been published [21]. Measurements were carried out bilaterally.

The Modified Ashworth Scale (MAS) is a 6 step ordinal scale [28] and is commonly used in clinical practice and clinical research. The MAS scale does not separate mechanical and neural contributions to passive movement resistance in a spastic muscle [29]. Although the MAS has several recognized limitations, it is widely used and enables assessments of both upper and lower extremities. Thus, spasticity according to the MAS (range 0 (no spasticity) to 5 (rigidity)) was assessed at M1 and M2 in both upper- (internal rotators of the shoulder, extensors and flexors of elbow, pronators and supinators of forearm and flexors of the wrist, and fingers) and the lower extremity (hip adductors, knee flexors and extensors, ankle plantar flexors (soleus and gastrocnemius and) and supinators of the ankle). Results are presented for the flexors of the wrist and were summarized for the upper extremity (maximum score 35p) and lower extremity (maximum score 30p). Measurements were carried out bilaterally.

#### Additional assessments of functioning and disability performed at M1 and M2

To assess sensorimotor function in the upper and lower extremity, the Fugl-Meyer Assessment of motor recovery (FM) for the upper (FM-UE) and lower (FU-LE) extremities were used (including motor-, sensory- and passive joint function and pain rated on a 3 point scale) [30]. Grip strength (kilogram) was measured by use of a digital hand dynamometer (www.Saehan.com). The Action Research Arm Test (ARAT) [20] was used as an observational rating scale of upper extremity performance including a 4 point scale assessing the performance of grasp, grip, pinch and gross movement. Walking speed was assessed with the 10 m walk test [31] (for walking speed in seconds). The 6 min walk test was used to test endurance (in meters) [32]. Balance was assessed by means of the Berg Balance Scale (14 items including static and dynamic movements rated on 5-point scales) [33, 34]. Finally, the participants’ perception of functioning and disability was assessed with the Stroke Impact Scale (SIS) where each question is responded to using a 6-point scale and a summary score for each of the 7 domains is calculated by use of an algorithm [35, 36].

### Statistics

Descriptive statistics was presented as mean and standard deviation (SD) for normally distributed continuous data and as median and interquartile range (IQR) for ordinal and not normally distributed data (detected with the Shapiro-Wilk test). To assess differences between more than two timepoints, Related Samples Friedman’s Two-Way Analysis was used for not normally distributed continuous data. To assess differences between baseline and after the intervention Paired Samples T-test was used for normally distributed continuous data and Related Samples Wilcoxon Signed Rank Test was used for approximately symmetric differences and the Sign Test for not normally distributed data. Bonferroni correction was used for correction for multiple comparisons. To assess correlation between ordinal data and continuous data, Spearman’s rho was used. Correlation coefficients < 0.25 were considered as little to no, 0.25–0.50 as fair, 0.50–0.75 as moderate to good, and > 0.75 as good to excellent association [37]. To explore to what extent a change in continuous data could be explained by the baseline values, a univariate linear regression analysis was performed for data with a normal distribution of residuals. Analyses were two-tailed and level of significance was set to *p* < 0.05. There was full adherence to the study protocol except for one participant with missing data for the 3 initial assessments (A1-A3) of the quantified NC as a contributor to spasticity. This participant was excluded from the A1-A3 analysis.

## Results

### Inclusion

A total of 30 persons were tested for eligibility. Nine were excluded as no neural component exceeding the cut off for spasticity according to the NeuroFlexor™ (> 3. 4 Newton) in the wrist flexors could be detected and 1 person due to wrist contractures prohibiting testing. Characteristics of the include participants are presented in Table 1.

**Table 1:** Characteristics of the included participants (*n* = 20)

Age, mean (SD) range	58.05 (12.86) 28–79
Women/Men, n	7/13
Infarction/haemorrhagic, n	11/9
Paretic side right/left, n	10/10
Time to inclusion, months, mean (SD) range	67.20 (44.30) 19–172
Independence in walking, Functional Ambulation Categories, (0-5p)^a^ median (IQR) range	4.50 (1.00) 2–5
Self-care and mobility, Barthel Index (0-100p)^a^, median (IQR) range	95.00 (25.00) 40–100
Cognitive function, Montreal cognitive assessment, (0-30p)^a^ mean (SD) range	21.45 (4.22) 12–28

^a^minimum and maximum points of the score (a lower score indicates increased impairments/limitations)

### Usability and perceived effects

According to the weekly telephone interviews, the participants used the suit 19.25 times in mean (SD 2.4, range 12–21 times). Two participants did not use the suit during the last week before follow-up due to hospitalization (*n* = 1) and travel (*n* = 1). During stimulation 8 participants were resting (sat or lay down), 10 were resting or moving (e.g. walking, performing ADL or exercising) and 2 only moved during stimulation. Overall usability of the suit was rated as 4.9 in mean (SD 2.4, range 0–9).

Perceived positive effects (*n* = 9) or possible positive effects (*n* = 3) on functioning was experienced by 12 (60%) of the included participants. Among the 12 participants, 6 experienced positive effects in both upper and lower extremity as well as a general effect, 3 only in the upper extremity and 3 only in the lower extremity. Positive effects were most commonly related to a perceived decrease in muscle tone (*n* = 9), improvements in gait pattern functions (*n* = 7) and control of voluntary movement in the upper extremity (*n* = 6). Eight participants did not perceive any positive effects on functioning. Disadvantages was reported by 9 participants and most commonly related to problems with putting on the suit (*n* = 8). One participant reported perceived limitations in mobility during stimulation and 1 participant reported gradual decrease in strength during the intervention period. Adverse events, reported by 3 participants were related to a tickling sensation in the sole of the foot during stimulation (*n* = 1), muscle soreness (like after strenuous exercise, *n* = 1) and an increase in muscle tone during the night (*n* = 1).

### Spasticity

**Fig. 3:**
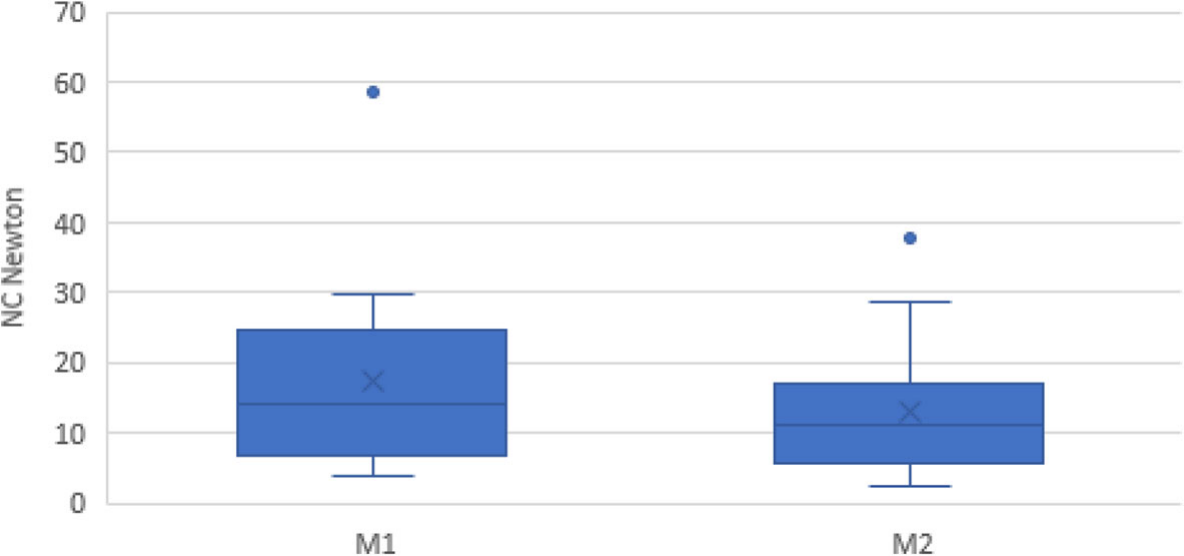
Neural component (NC) before (M1) and after the 6 weeks intervention (M2)

Analysis of the 3 initial assessments (A1-A3) of the quantified NC as a contributor to spasticity of the wrist flexors of the affected hand performed on 19 of the 20 included participants before the start of the intervention, showed no significant difference between assessments (Newton, median (IQR): A1: 13.15 (9.97), A2: 11.63 (11.70), A3: 11.68 (16.45), *p* = 0.949). The test for potential, immediate changes of NC during one treatment session performed before the intervention showed a decrease in the NC among 10 of the 19 included participants (median (IQR) -2.07 (3.35) Newton), while an increase was found among the remaining 9 participants (median (IQR) 1.42 (7.04) Newton). On a group level no significant difference in the NC during treatment compared to before treatment was found (*p* = 0.747). However, for the same 19 participants, a significant decrease in the NC was seen after the 6 weeks intervention period including repeated treatments, based on M1 and M2 data, (Newton, mean (SD): M1: 13.58 (18.15), M2: 10.89 (11.92), *p* = 0.040) and for the total of the 20 participants (*p* = 0.012) (Table 2 and Fig. 3). A total of 60% of the variance in the difference in NC of the affected hand could be explained by the baseline NC value (R square = 0.600, *p* = 0.000, B = − 0.418, CI: − 0.587; − 0.249). Three participants had a neural component > 3.4 Newton in the less affected hand, indicating bilateral spasticity. For the less affected hand no significant difference between M1 and M2 was found (Table 2). Change in the NC for the wrist flexors of the affected hand did not correlate significantly with perceived effect on functioning in the upper extremity (no/possible/perceived effect) (*r =* 0.240 *p* = 0.308).

**Table 2:** NeuroFlexor™ components and MAS at M1 and M2

Assessment	M1	M2	*P*-value
NeuroFlexor™, wrist flexors, affected hand, NC ^a^, Newton, median (IQR))	14.09 (17.71)	11.30 (11.27)	0.023
NeuroFlexor™, wrist flexors, less affected hand NC^a^, Newton, median (IQR)	1.10 (2.51)	0.69 (1.16)	0.314
MAS, wrist flexors, affected hand (0–5 p)^b^ median (IQR)	0.00 (2.00)	1.00 (2.00)	0.319
MAS, wrist flexors, less affected hand (0–5 p)^b^ median (IQR)	0.00 (0.00)	0.00 (0.00)	N/A¨
MAS-sum, Upper extremity affected side, summarized score (0–35 p)^b^ median (IQR)	9.00 (7.00)	9.00 (8.00)	0.354
MAS-sum, Upper extremity less affected side, summarized score, (0–35 p)^b^ median (IQR)	0.00 (0.00)	0.00 (0.00)	0.317
MAS-sum, Lower extremity affected side, summarized score, (0–30 p)^b^ median (IQR)	7.00 (9.00)	5.00 (3.00)	0.273
MAS-sum, Lower extremity less affected side, summarized score, (0–30 p)^b^ median (IQR)	0.00 (2.00)	0.00 (1.00)	0.250

^a^NC > 3.4 Newton was considered as hand spasticity (Pennati 2016), ^b^Summarized minimum and maximum points (no spasticity to rigidity), ¨*n* = 1 participant with detected spasticity

Spasticity according to the MAS was detected at M1 and M2, in both upper and lower extremities in all participants (Table 2). One participant was affected bilaterally in the upper extremity and 7 participants in the lower extremity according to the summarized score of the MAS (MAS-sum score). No significant change between M1 and M2 for the wrist flexors of the affected hand was found according to the MAS (*p* = 0.319) nor for the MAS-sum scores for the upper (affected side *p* = 0.354, less affected side *p* = 0.317) and lower extremities (affected side *p* = 0.272, less affected side *p* = 0.250) (Table 2).

### Additional assessments of functioning and disability

Results of assessed function and activity in the upper and lower extremities are presented in Tables 3 and 4.

**Table 3:** Function and activity at M1 and M2 in the affected upper extremity

Assessments	M1	M2	*P*
FM-UE, total score (0-126p)^a^ mean (SD)	63.90 (7.99)	66.55 (7.86)	0.000
FM-UE, motor score^ (0–66 p)^a^ mean (SD)	29.80 (14.70)	33.60 (16.55)	0.000
FM-UE, sensory function, (0-12p)^a^ median (IQR)	5.50 (9.00)	7.50 (10.00)	0.032#
FM-UE, passive joint motion, (0–24)^a^ median (IQR)	20.00 (4.00)	21.50 (4.00)	0.004
FM-UE, pain, (0-24p)^a^ median (IQR)	24.00 (2.00)	24.00 (2.00)	0.719
ARAT total score (0-57p)^a^ median (IQR)	24.50 (28.50)	25.00 (28.75)	0.000
JAMAR, kilograms mean (SD)	9.15 (4.94)	9.27 (5.10)	0.844

^a^minimum and maximum points of the score (a lower score indicates increased impairment) # not significant after a Bonferroni correction including the FM- UE comparisons

**Table 4:** Function and activity at M1 and M2 involving the affected lower extremity

Assessments	M1	M2	*P* value
FM-LE, total score (0-86p) ^a^ mean (SD)	63.90 (7.99)	66.55 (7.86)	0.003
FM-LE, motor score (0-34p) ^a^ mean (SD)	19.60 (5.68)	21.70 (5.45)	0.001
FM-LE, sensory (0-12p) ^a^ mean (SD)	7.25 (3.74)	7.30 (3.72)	0.874
FM-LE, passive joint motion, (0-20p) ^a^ median (IQR)	18.00 (3.00)	19.00 (3.00)	0.013#
FM-LE, pain, (0-20p)^a^ mean (SD)	20.00 (1.00)	20.00 (1.00)	0.527
Berg balance scale (0-56p) ^a^ median (IQR)	46.00 (13.00)	49.50 (9.00)	0.063
10 m walk test, seconds, mean (SD)	22.22 (28.77)	21.76 (25.93)	0.629
6 min walk, meters, mean (SD)	252.88 (153.44)	255.50 (155.60)	0.746

^a^minimum and maximum points of the score (a lower score indicates increased impairment)

# not significant after a Bonferroni correction including the FM- LE comparisons

Change between M1 and M2 in the FM total scores for the affected upper and lower extremity respectively could not be significantly explained by baseline FM total score (FM-UE: R square = 0.069, *p* = 0.263, B = 0.069, CI: − 0.056; 0.194) (FM-LE: R square = 0.066, *p* = 0.276, B = − 0.113, CI: − 0.323; 0.098). However, change between M1 and M2 in the FM-UE total score correlated significantly and fairly with perceived effect in the upper extremity (*r* = 0.498, *p* = 0.025) and in the corresponding analysis for the change in the total score of the FM-LE and the perceived effect in the lower extremity (*r =* 0.469, *p* = 0.037). No significant correlation was found between the change between M1 and M2 in the ARAT total score of the affected extremity and perceived effect in the upper extremity (correlation coefficient *r* 0.123, *p* = 0.606).

### Self-perceived rating of functioning and disability

Results of self-perceived rating of functioning and disability according to the Stroke Impact Scale is presented in Table 5. No significant change was found between M1 and M2.

**Table 5:** Self-perceived ratings of functioning and disability according to the Stroke Impact scale at M1 and M2 and test of significant differences between M1 and M2

Domains^a^	M1	M2	*P* value
1.Strength, mean (SD)	33.75 (13.96)	39.68 (10.97)	0.073
2.Memory and thinking, mean (SD)	78.93 (14.74)	80.18 (13.73)	0.612
3.Emotion, mean (SD)	72.08 (16.75)	71.94 (17.50)	0.947
4.Communication, median (IQR)	87.50 (16.07)	85.71 (9.82)	0.219
5.ADL, mean (SD)	65.90 (21.97)	66.67 (21.51)	0.618
6.Mobility, mean (SD)	74.86 (18.21)	76.39 (18.28)	0.346
7.Hand function, median (IQR)	0.00 (35.00)	10.00 (38.75)	0.301
8.Social Participation, mean (SD)	54.53 (29.48)	55.78 (23.54)	0.775
9.Recovery^b^, mean (SD)	50.05 (24.72)	53.30 (23.99)	0.390

^a^Each domain ranges from 0 to 100 (0 = maximum limitation and restriction to 100 = no limitation or restriction). ^b^Rated on a vertical scale, ranging from 0 = no recovery to 100 = full recovery

## Discussion

This study targets a subgroup of the stroke population living with long-term consequences of hemiparetic stroke including spasticity, impaired sensorimotor function, and activity limitations. The aim of this study was to explore if assessed and perceived functioning and disability was affected by using the Mollii® suit for treatment of spasticity in the home setting for 6 weeks. In line with previously reported results from the same study setting [38], approximately 2/3 of the screened eligible participants were found to have a clinically detectable neural component exceeding the cut off for spasticity according to the NeuroFlexor™. Thus, the participants included in this study may be considered representable for a slightly younger stroke population (mean age 58 years), living with hemiplegia and spasticity long-term after stroke.

Main findings of this study include a significant decrease in NC of the wrist flexors detected with the NeuroFlexor™. Manual assessments of upper and lower extremities according to the Ashworth scale however, did not show a significant change. Sensorimotor function in both upper and lower extremities improved significantly according to the FM total scores while assessed activity performance did not, apart from observed grasp, grip, pinch and gross movements assessed with the ARAT. Perceived positive effects on functioning were reported by 60% and, in line with results from the clinical assessments, effects were most commonly related to a perceived decrease in muscle tone, improvements in gait pattern functions and/or control of voluntary movement in the upper extremity. In addition, perceived positive effects were found to be correlated with improvements in sensorimotor function according to the FM total scores, indicating that perceived effects described by patients may correspond with clinical measures of sensorimotor function. The study is explorative and may guide future controlled studies needed to confirm these results.

Compliance and perceived effects were thoroughly monitored by structured weekly telephone interviews. Overall, compliance to study instructions was high. The suit was used in mean 19.25 out of potentially 21 times indicating that use of the Mollii® suit in this patient population is feasible, which agrees with a previous study [18]. Nevertheless, mean ratings of overall usability were modest and the main reported perceived disadvantage was related to problems with putting on the suit. These results indicate that further development of the suit may include a more flexible fabric and design to ease the donning without compromising the tight fit needed to adhere the electrodes to the skin surface.

In this study we used the standardized evaluated NeuroFlexor™ method to quantify and differentiate the neural, i.e. spasticity, elastic and viscosity components of an increased resistance to passive stretch [21, 25–27, 38]. The observation of stable baseline the NC values before the start of the intervention (A1-A3) lends strong support to the observed significant changes in the NC between the start (M1) and the end of the intervention (M2). While repeated baseline measurements (A1-A3) showed no significant variation, spasticity was significantly lower after the 6 weeks intervention and 60% of the variance in the difference in the neural component of the affected hand could be explained by the baseline NC value, indicating that the effect on the NC will be larger if the NC is high at baseline.

Notably, only the wrist flexors were assessed with the NeuroFlexor™ hand module. This is a limitation in a study assessing effects in both upper and lower extremities. Therefore, assessments with the MAS was performed. These results exhibited no significant change after the intervention although the most commonly reported positive effect was a decrease in muscle tone. These results may reflect that the MAS has low accuracy for diagnosing reflex-mediated resistance to passive stretch [39, 40]. A recent study assessing the validity of the NeuroFlexor™ and the MAS confirms that clinical assessment according to the modified Ashworth scale cannot differentiate the active NC from the passive elastic component [41]. A new NeuroFlexor™ foot module has been developed and is currently tested on a stroke population and in healthy subjects.

In the current study, 3 participants had a detected neural component above cut-off (> 3.4 Newton) in the less affected hand, and 7 participants had increased resistance to passive stretch in the lower extremity according to the Ashworth scale, indicating bilateral affection. These findings are in agreement with previous studies demonstrating that motor function on the ipsilesional side may also be affected due to an interhemispheric imbalance after stroke [38, 42]. Taking the questioned validity of the MAS into consideration [41], the bilateral findings based on assessment with the modified Ashworth scale could also be a result of a higher resistance in the elastic component rather than a neural.

Given the observed results on sensorimotor function and upper extremity performance, future studies should move on to evaluate the effect of combining treatment with the Mollii® suit with rehabilitation interventions targeting activity performance. In clinical practice, treatment of spasticity (both pharmacological and non-pharmacological) is strongly recommended to be combined with a multidisciplinary approach with goals set at an activity and participation level [43, 44]. As presented in this study, a decrease in NC was detected in 50% of the participants while resting in a sitting position during a single treatment with the Mollii® suit. However, there was no significant decrease of NC on a group level in this test situation while a significant decrease in the NC was seen after recurrent treatments over 6 weeks when 60% performed everyday life activities in association with treatments with Mollii. This reasoning is supported by positive results from a systematic review on treatments of spasticity using TENS during activity [14]. Thus, both repetition of treatments and that treatment is performed during activity may be contributing factors and explain why NC did not decrease significantly on a group level during one treatment session performed at rest.

Future studies should also consider a double blinded design where the suit is used turned on or off to explore this further. The electric stimulation given by the suit and its effect on spasticity was the focus of this study, still the full body compression that the suit provides is a factor that may be considered. This could potentially increase the tactile and proprioceptive input, factors that are known to be important for motor recovery after stroke [45].

## Conclusion

This is the first study that demonstrates the feasibility of regular use of the Mollii® method in the home setting and potentially beneficial effects after a 6 weeks intervention on spasticity and sensorimotor function in patients with chronic stroke. Results need to be confirmed in a larger controlled study preferably combined with goal directed training interventions to enhance possible effects on activity and participation domains.

## Data Availability

The datasets generated and/or analyzed during the current study will be shared upon reasonable request. All data are included in this published article.

## Abbreviations

M1: Assessment before the intervention
M2: Assessment after the 6 weeks intervention
ARAT: Action Research Arm test
EC: Elastic component
FM: Fugl-Meyer Assessment of motor recovery
FM-UE: Fugl-Meyer Assessment for the upper extremities
FM-LE: Fugl-Meyer Assessment for the lower extremities
IQR: Interquartile Range
MAS: Modified Ashworth scale
MAS-sum: Modified Ashworth scale summarized score
NC: Neural component
SD: Standard deviation
TENS: Transcutaneous electrical nerve stimulation
